# The continuing discovery on the evidence for RNA editing in SARS-CoV-2

**DOI:** 10.1080/15476286.2023.2214437

**Published:** 2023-05-18

**Authors:** Xiaoxin Pu, Qinwei Xu, Jinxiang Wang, Baoyi Liu

**Affiliations:** a Department of Respiratory and Critical Care Medicine, Qilu Hospital (Qingdao), Cheeloo College Medicine, Shandong University, Qingdao, China; b Department of Respiratory and Critical Care Medicine, Qilu Hospital, Cheeloo College Medicine, Shandong University, Jinan, China

**Keywords:** Evidence, C-to-U RNA editing, SARS-CoV-2, evolution, APOBEC motif

## Abstract

Recent studies have presented strong evidence that C-to-U RNA editing is the driving force that fuels severe acute respiratory syndrome coronavirus 2 (SARS-CoV-2) evolution. The findings finally ended the long-term debate on the evolutionary driving force behind SARS-CoV-2 evolution. Here, we would first acknowledge the breakthroughs made by the recent works, such as using the global SARS-CoV-2 data to demonstrate the major mutation source of this virus. Meanwhile, we would raise a few concerns on the accuracy of their interpretation on C-to-U RNA editing. By re-analysing the SARS-CoV-2 population data, we found that the editing frequency on C-to-U sites did not perfectly correlate with the binding motif of the editing enzyme APOBEC, suggesting that there might be false-positive sites among the C-to-U mutations or the original data did not fully represent the novel mutation rate. We hope our work could help people understand the molecular basis underlying SARS-CoV-2 mutation and also be useful to guide future studies on SARS-CoV-2 evolution.

## 1. Our current knowledge on the mutation source of SARS-CoV-2

There is a continuous concern on the fast evolution of severe acute respiratory syndrome coronavirus 2 (SARS-CoV-2), as the new strains might escape the current vaccines and make our efforts futile. Thus, understanding the molecular basis underlying SARS-CoV-2 mutation and evolution is essential. Recent findings have provided strong evidence that the rampant C-to-U RNA-editing events are the major sources of mutations in SARS-CoV-2 [1,2]. This was the first to systematically look into the mutation profile obtained from global SARS-CoV-2 population database (millions of sequences) [3] and meanwhile taking the allele frequency (AF) messages into account.

This breakthrough was made after a long-term debate on the origin of mutation source of the virus. However, despite the seemingly perfect evidences for viral RNA editing [1,2], there are still some additional concerns that shed doubts on the authenticity of those C-to-U RNA-editing events.

Here, we will (i) briefly introduce the debate on the mutation source of SARS-CoV-2: how the debate began, progressed and ended and then (ii) raise a few concern to show that the current evidences for C-to-U RNA editing in SARS-CoV-2 are not 100% solid. Therefore, maybe the debate has not been completely ended yet.

## 2. The original debate: what and why?

There is a long-term debate on the major driving force that accelerates the evolution of SARS-CoV-2 [4]. Based on molecular biology, the numerous single nucleotide variants (SNVs) found in the SARS-CoV-2 RNAs come from two resources: (i) the replication error introduced by RNA-dependent RNA polymerase and (ii) RNA-editing events mediated by the host’s deamination enzymes (ADARs for A-to-I editing and APOBECs for C-to-U editing). The main point of the debate is which resource is the major contributor of the mutations in SARS-CoV-2.

For DNA organisms like humans, the replication errors produce DNA mutations like SNPs while RNA-editing enzymes produce variations in RNAs. The two resources would be highly distinguishable if one performs DNA resequencing plus a matched set of RNA sequencing [5]. However, for RNA viruses like SARS-CoV-2, there is no DNA genome so that the replication induced SNPs and the RNA-editing events are ‘entangled’ in the viral RNA molecules. This fact adds difficulty to the debate on replication-error-origin *versus* RNA-editing origin of the mutations in SARS-CoV-2 [6]. For a given SNV (say, A-to-G mutation) in the viral sequence, one could not easily tell whether this SNV came from a replication A-to-G error or from A-to-I RNA editing (note that I is read as G). The same goes for C-to-T replication error *versus* C-to-U RNA editing [7].

## 3. The progression of the debate: is there evidence for RNA editing in SARS-CoV-2?

**Figure 1.**
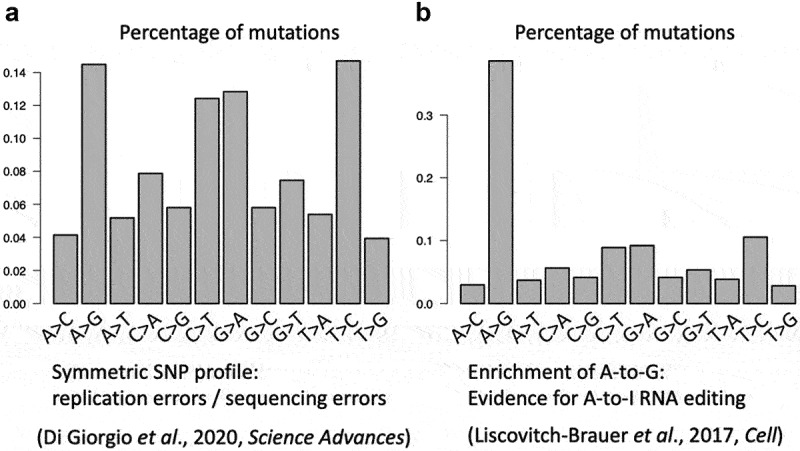
Examples of the mutation profile. (a) A symmetric mutation profile typically suggests that these mutations come from replication errors or sequencing errors [8]. (b) An enrichment for a particular mutation type like A-to-G is evidence for RNA editing [9].

An initial attempt to identify SNVs in the SARS-CoV-2 transcriptome has discrepancy between its results and conclusions [8]. Its results support the replication-error-origin of the viral mutations (because it shows a symmetric SNV landscape) (Figure 1A), but its conclusion turns out to say that RNA-editing events are abundant in SARS-CoV-2 [8]. Since there are obvious logical flaws in the interpretation of Giorgio et al.’s [8] paper, it becomes confusing whether replication errors or RNA-editing events are the driving force of SARS-CoV-2 evolution.

Not surprisingly, multiple papers [10–13] were published to criticize the Giorgio et al.’s [8] paper from different aspects. However, none of these critical papers have carried out a much better methodology to tell people how confidence we are to find reliable RNA-editing events in SARS-CoV-2.

A major obstacle is the difficulty to enrich a particular type of SNV (such as A-to-G, Figure 1B) [9,14,15], rather than simply providing a symmetric SNV landscape that resembles the SNP distribution produced by replication errors (Figure 1A). While Giorgio et al. [8] failed to prove RNA editing, the follow-up papers did no better (at least not significantly improved as we see) [10–13]. Here comes a dilemma: if strong evidence for RNA-editing events could not be provided, then the replication-error-origin of viral mutations as shown by Giorgio et al. [8] will be accepted by people.

## 4. The debate ends with C-to-U RNA editing

This debate finally ends with an observation that the majority of the mutations in the global SARS-CoV-2 population are C-to-T (weighted by allele frequency) [1,2], suggesting that C-to-U RNA-editing events are the major contributors of the mutations in SARS-CoV-2. This should be the first *in silico* evidence for RNA editing in SARS-CoV-2 by using such a large scale of data.

Apart from the abundance of C-to-T mutations, there were other evidence for the existence of C-to-U RNA editing. It is known that the C-to-U editing enzyme APOBEC prefers single-stranded RNA. Accordingly, researchers observed that the allele frequency of C-to-T mutation increases with the confidence of single-stranded RNA suggested by *in vivo* measurement [16]. If the C-to-T mutations came from replication errors, then they should not have a preference on RNA structure. The only plausible explanation for the correlation between C-to-T occurrence and single-stranded RNA structure is that these variations are C-to-U RNA-editing events.

Another advantage in proving the editing-origin of viral mutation was that only the synonymous mutations were used for the RNA structure analysis [2]. Missense mutations are suppressed by natural selection so that their allele frequency may not accurately reflect the preference of RNA editing enzymes.

At this stage, it seems that the debate on the mutation source in SARS-CoV-2 is ended. Although the initial attempt to identify SNVs in SARS-CoV-2 [8] was questionable, after two years of investigation, now it is highly acknowledged that C-to-U RNA editing dominates the mutations in SARS-CoV-2 population.

## 5. New concerns regarding the authenticity of C-to-U RNA-editing sites

Both C-to-U and A-to-I RNA-editing events not only have preference on RNA structure but also have preference on sequence context. For A-to-I editing, ADAR favours an adenosine in a ‘nonG+Adenosine+G’ motif [17] (although with some exceptions that differ from the traditional ADAR binding motif). This means that the adenosines after a non-G should have higher editing frequencies than the adenosines after a G, and the adenosines before a G should have higher editing frequencies than the adenosines before a non-G.

Researchers directly applied the ADAR-binding motif to the APOBEC-binding motif and found that C-to-U RNA-editing frequencies (allele frequencies of C-to-T mutations) are correlated with the optimality of sequence context [2]. Then, it is intuitive to believe that this consolidated the RNA-editing-origin of these rampant C-to-T mutations.

We repeated the data by downloading the time-series mutation profile of global SARS-CoV-2 population [3,18,19]. The mutation frequency of a site was calculated as the number of sequences supporting the mutation (alternative allele) divided by the total number of sequences covering this site. The nucleotide frequency (proportion) upstream or downstream the C-to-U sites was calculated by extracting the nucleotide positions before or after all the C-to-U positions.

**Figure 2.**
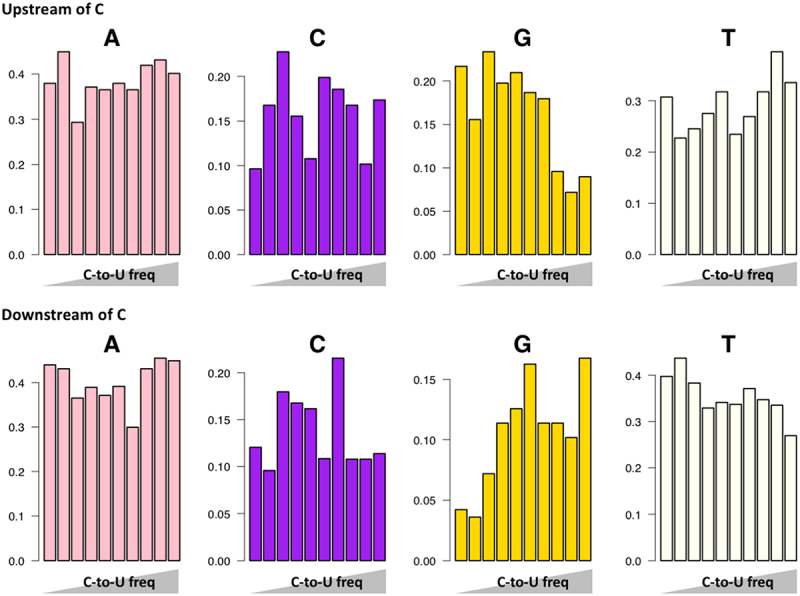
Synonymous C-to-T mutations, representing C-to-U RNA editing, were ranked by their allele frequency in SARS-CoV-2 population. Allele frequency would represent RNA-editing frequency. The percentages of the upstream or downstream nucleotides were displayed.

We indeed found that the fraction of an upstream G decreases with C-to-U editing efficiency, while the fraction of a downstream G increases with C-to-U editing efficiency (Figure 2). This phenomenon agrees with previous observation that an upstream G is unfavourable while a downstream G facilitates C-to-U editing [2].

However, the binding motif of APOBECs is slightly different from that of ADARs as reported by base editor studies [20]. It is said that for C-to-U RNA-editing sites the upstream preference is adenosine while the downstream preference is thymidine (uridine) [20]. Note that both DNA and RNA could be targeted by APOBECs with different binding motifs and the targeting efficiency is even affected by the DNA/RNA structures [21,22]. Here, we only focused on the RNA-editing role of APOBECs and intriguingly we did not observe preference on upstream adenosine for the highly edited C-to-U sites and we even observed a slight decrease of downstream T for the highly edited C-to-U sites (Figure 2). These two results disagree with the APOBEC preference reported by Grunewald et al. [20].

This does not mean that Liu et al. [2] failed to provide evidence for C-to-U RNA editing in SARS-CoV-2. They already showed a striking enrichment of C-to-T mutations, and this should be the most powerful evidence. The correlation between C-to-T frequency and single-stranded RNA structure further consolidated the existence of C-to-U RNA-editing events. The only stain is that the sequence context does not fit the experimentally examined preference of APOBEC [20].

## 6. Explanations for the context issue and future perspectives

Possibly, the major determinant for APOBEC editing on SARS-CoV-2 is the RNA structure rather than nucleotide context [23]. This assumption could be tested by artificially introducing point mutations to SARS-CoV-2 sequences to alter the context of known C-to-U editing sites. Alternatively, another explanation is that the allele frequency in global SARS-CoV-2 population does not represent the novel mutation rate within a short period. If one inspects the novel mutations within a short time window and calculates novel mutation rate, one might obtain strong correlation between C-to-T(U) mutation rate and the sequence context of editing sites.

In summary, the debate on the source of mutations in SARS-CoV-2 lasts for 2 years and now it almost ends up with an agreement that rampant C-to-U RNA editing is the major source of SARS-CoV-2 mutation. The host-dependent RNA editing fuels the evolution of SARS-CoV-2. Therefore, to get control of the mutation and the evolution of SARS-CoV-2, cutting down the virus transmission is still an efficient way [24]. Understanding the molecular mechanisms underlying SARS-CoV-2 mutation and evolution is very helpful for our control of the pandemic.

## Data Availability

All data used in this study were downloaded from a previous study [2] https://rnajournal.cshlp.org/content/28/7/917, the data of which were originally generated by GISAID [3,19] https://gisaid.org/.
